# Species of the subgenus Empis (Xanthempis) from South Korea (Diptera, Empididae)

**DOI:** 10.3897/zookeys.769.24545

**Published:** 2018-06-26

**Authors:** Jiale Zhou, Igor Shamshev, Yongjung Kwon, Ding Yang

**Affiliations:** 1 Department of Entomology, College of Plant Protection, China Agricultural University, Beijing 100193, China; 2 Zoological Institute, Russian Academy of Sciences,; 3 Universitetskaya nab. 1, St. Petersburg, 199034, Russia; 4 School of Applied Biosciences, College of Agriculture & Life Sciences, Kyungpook National University, Daegu 702-701, South Korea

**Keywords:** Diptera, Empididae, new species, South Korea, *
Xanthempis*

## Abstract

The subgenus Empis (Xanthempis) is newly recorded from South Korea with the following two species: E. (X.) sesquata (Ito, 1961) and E. (X.) suhi **sp. n.** A key to the known species of *Xanthempis* from Eastern Asia is presented. The distribution of *Xanthempis* in eastern Asia is briefly discussed.

## Introduction

Xanthempis Bezzi, 1909 is a small subgenus of the genus Empis Linnaeus, 1758 with 52 known species, which are distributed only in the Palaearctic region (Yang et al. 2007, Shamshev and Kustov 2008, Daugeron 2009, Kustov 2011, Daugeron and Lefebvre 2015). The subgenus is characterized by a relatively large size (4–8 mm), yellow coloration of the body, posterior elongation of the head, relatively small eyes separated by a wide frons in both sexes, long antennal scape, strong reduction of the body chaetotaxy, and some other features (Chvála 1996, Shamshev and Kustov 2008). The Palaearctic species were studied by Chvála (1994, 1996), Daugeron (1997, 2000, 2009), Shamshev (1998), Shamshev and Kustov (2008) and Daugeron and Lefebvre (2015). Shamshev (1998) compiled a key to all species of the subgenus. Later, Shamshev and Kustov (2008) keyed the species of *Xanthempis* found from the Caucasus, including a species described from Iran, and Daugeron (2009) provided a key to Pyrenean species. The swarming and mating behaviors of *Xanthempis* species were reported by Plant (1994) and Preston-Mafham (1999).

In the present paper, two species are added to the fauna of South Korea, one of which is described as new to science. A key to the known species of the subgenus Xanthempis from eastern Asia is presented, mainly based on Shamshev (1998).

## Materials and methods

Specimens used for our study are deposited in the Insect Collection of Kyungpook National University, Daegu, South Korea. Terminalia preparations of males were made by macerating the apical portion of the abdomen in cold 10% NaOH for 12–15 hours. After examination, the preparations were transferred to fresh glycerine and stored in microvial pinned below the dry-pinned specimen, and the descriptions are based on dry-pinned material. Morphological terminology for adult structures mainly follows McAlpine (1981), and the structures of the male genitalia follow Cumming and Wood (2009). Photographs were taken with a Digital Microscope VHX-1000 series at China Agricultural University and then stacked by Helicon Focus 6.0. The following abbreviations are used:

**acr** acrostichal seta(e),

**d** dorsal seta(e),

**dc** dorsocentral seta(e),

**npl** notopleural seta(e),

**oc** ocellar seta(e),

**ppn** postpronotal seta(e),

**prsc** prescutellar seta(e),

**pa** postalar seta(e),

**sa** supraalar seta(e),

**sc** scutellar seta(e).

## Taxonomy

### Key to species (males) of subgenus Xanthempis from Eastern Asia

**Table:** 

1	Mesoscutum entirely yellow	**Empis (Xanthempis) kovalevi Shamshev**
–	Mesoscutum with brownish to blackish longitudinal vittae and sometimes also with spots	**2**
2	Mesoscutum with broad median vitta running also over scutellum and postnotum and with elongate blackish spot between postpronotal lobe and suture	**Empis (Xanthempis) sesquata (Ito)**
–	Mesoscutum only with median vitta	**3**
3	Occiput with large rhomboid spot dorsally	**Empis (Xanthempis) stercorea Linnaeus**
–	Occiput with drop-like to wedge-shaped spot dorsally (including ocellar tubercle)	**4**
4	Mesoscutal vitta disappearing before prescutellar depression	**5**
–	Mesoscutal vitta running to base of scutellum	**6**
5	Mesoscutum (dorsal view) shiny, median vitta distinctly bordered; epandrial lamella broad (lateral view); hypandrium with small pointed apical prominence bearing 2 minute hair-like setae	**Empis (Xanthempis) japonica Frey**
–	Mesoscutum (dorsal view) faintly greyish pollinose, rather subshiny; epandrial lamella narrow (lateral view); hypandrium with large apical prominence bearing 2 spinules	**Empis (Xanthempis) richteri Shamshev**
6	Mesoscutal vitta very narrow, in front of suture occupying about 1/4 of space between rows of dc setae; phallus with dorsal projection on subapical part (lateral view)	**Empis (Xanthempis) suhi sp. n.**
–	Mesoscutal vitta broader, in front of suture occupying at least 1/3 of space between rows of dc setae; phallus smoothly curved on subapical part (lateral view)	**7**
7	Hypandrium with large subtriangular apical prominence (ventral view)	**Empis (Xanthempis) zlobini Shamshev**
–	Hypandrium with straight margin apically	**Empis (Xanthempis) belousovi Shamshev**

#### Empis (Xanthempis) sesquata

Taxon classificationAnimaliaDipteraHybotidae

(Ito)

Figs 1–2 , 3–6

Xanthempis sesquata Ito, 1961: 127, Abb. 6, 7. Type locality: Karyôsan (Aki) [now Hiroshima Prefecture], Japan (Honsyû).

##### Diagnosis.

Mesoscutum with broad black median longitudinal vitta running also over scutellum and postnotum and elongate blackish spot between postpronotal lobe and suture. Occiput with contrastingly black, wedge-shaped spot including ocellar tubercle. Prothoracic spiracle yellow.

##### Description.

**Figures 1–2.**
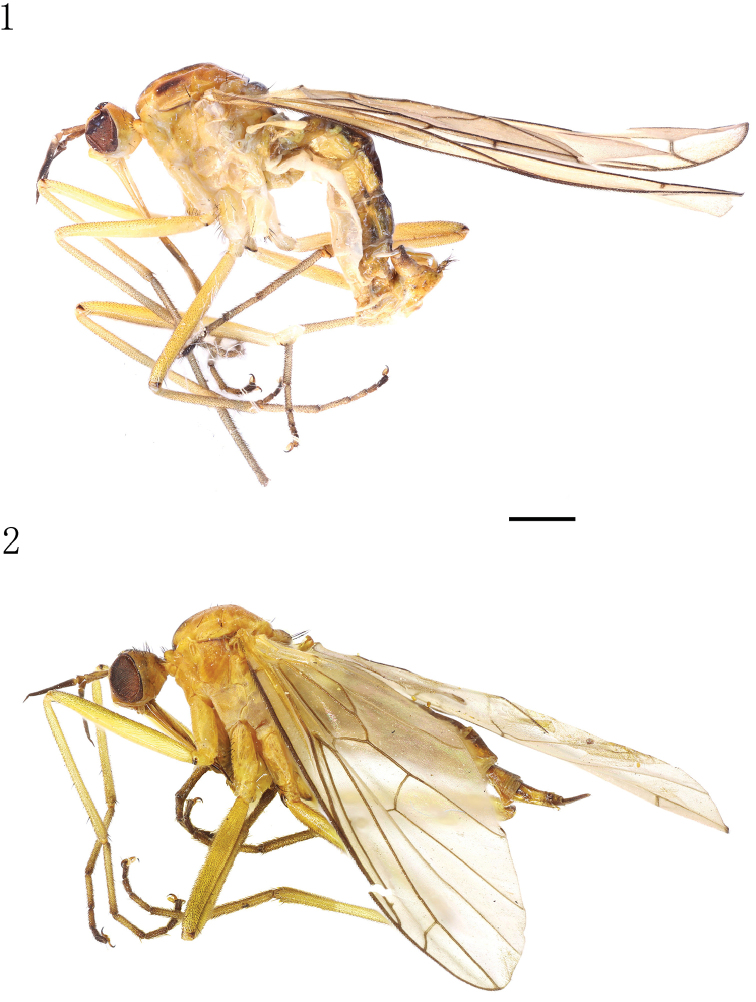
Empis (Xanthempis) sesquata (Ito), habitus, lateral view. **1** Male **2** Female. Scale bar: 1 mm.

Male (Fig. 1). *Body* length 6.0–6.1 mm, wing length 8.5–8.6 mm. Head (Fig. 4) largely brownish yellow or yellow, faintly pale greyish pollinose; frons and face dark yellow, occiput with narrow contrastingly black, wedge-shaped spot including ocellar tubercle. Eyes dichoptic, ommatidia equally small. Frons broad, parallel-sided, with minute dark setulae laterally. Occiput (Fig. 6) with short sparse black setae arranged in two almost regular transverse rows on upper part and some pale setae behind mouth-opening. Ocellar tubercle with two short proclinate oc and some minute setulae. Antennal scape and pedicel brownish, postpedicel and stylus black; scape long, approx. five times longer than wide, with some black setulae; pedicel very short, subglobular, with circlet of black subapical setulae; postpedicel very long, narrow, subconical, approx. eight times longer than wide; stylus very short, nearly 0.3 times as long as postpedicel. Proboscis long, labrum approx. two times longer than head height; palpus yellow, with scattered blackish setulae.

**Figures 3–6.**
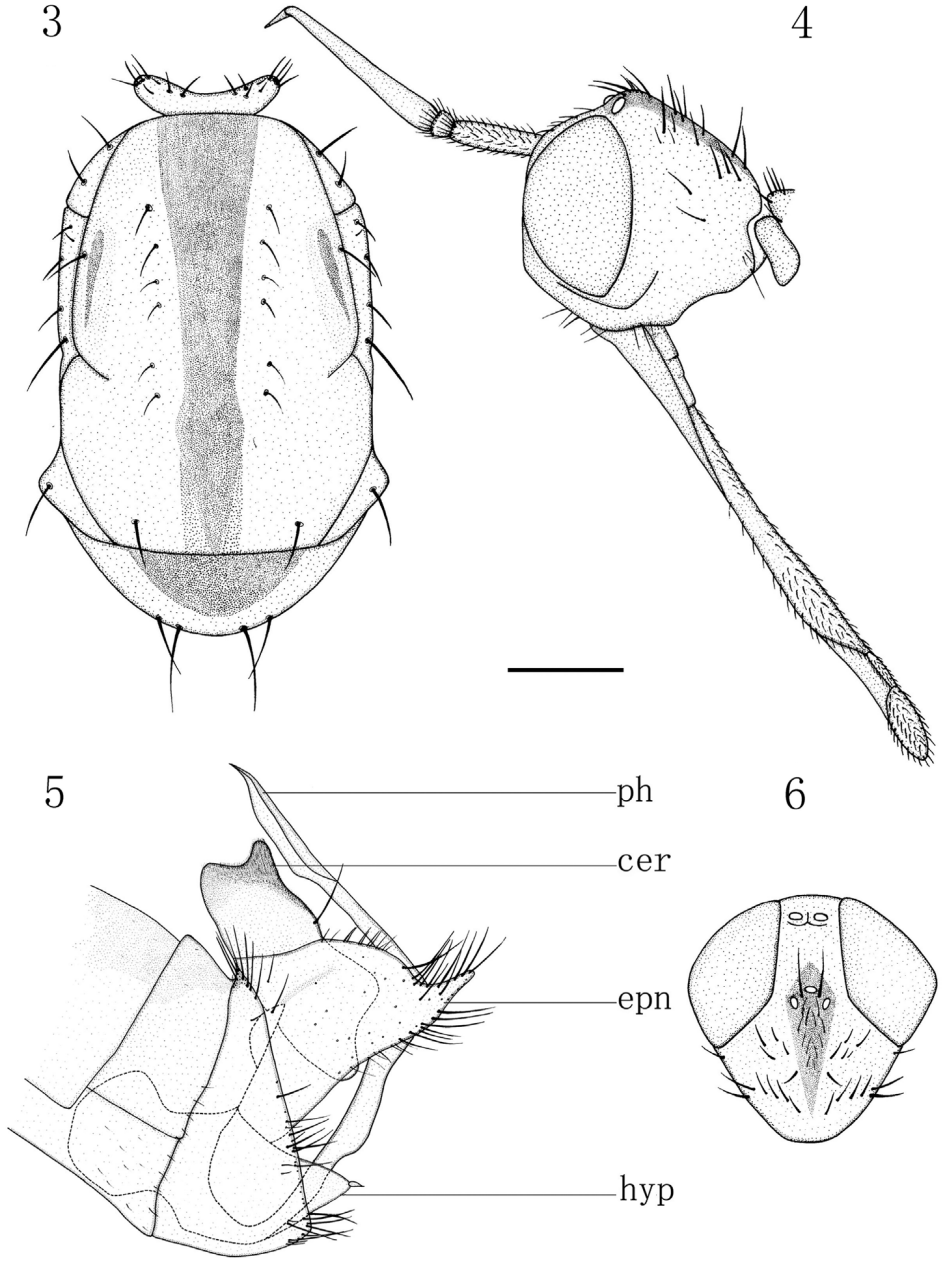
Empis (Xanthempis) sesquata (Ito), male. **3** Thorax, dorsal view **4** Head, lateral view **5** Genitalia, lateral view **6** Head, dorsal view. Scale bar: 1 mm. Abbreviations: ph = phallus, cer = cercus, epn = epandrium, hyp = hypandrium.

*Thorax* largely yellow, faintly pale greyish pollinose, with greatly reduced, only black setation; mesoscutum (Fig. 3) with broad, black, distinct, median longitudinal vitta running also over scutellum and postnotum, as well as with elongate, blackish, less distinct spot between postpronotal lobe and suture on each side; antepronotum with dark spot dorsally. Prothoracic spiracle yellow. Prosternum bare. Proepisternum with 2–3 hair-like setulae on lower part. Antepronotum with several spinule-like setulae on each side. Postpronotal lobe with one short inclinate ppn and additional shorter seta anteriorly. Mesonotum with one short thin presutural sa, one long npl, one short pa (sometimes with 1–2 additional setulae), four sc (apicals short, laterals minute); acr absent; dc uniserial, 6–7 in row, situated outside black median vitta, hair-like, minute (except 1–2 prsc). Laterotergite with several black setulae of different lengths. Legs yellowish to brownish yellow, only tarsomere 5 dark brown; lacking prominent bristles (except circlets of short subapical setae on tibiae). Wing nearly hyaline; brownish yellow stigma long, narrow; veins dark brown; basal costal bristle black, very short. Calypter yellowish, blackish fringed. Halter yellow.

*Abdomen* extensively yellow but tergites broadly brownish dorsally forming uniform vitta, subshiny; with scattered, mostly yellowish to brownish yellow setulae longer and darker on sternite 8 posteriorly. Hypopygium (Fig. 5) large, almost entirely yellow, only cerci narrowly brownish apically. Cercus rather large, with deep excision, dorsal arm long, broad, somewhat concave apically (lateral view), ventral arm short, finger-like; covered with dark minute setulae and bearing a moderately long seta on ventral arm. Epandrial lobe rather trapezoid, with upper posterior corner broadly rounded and lower posterior corner narrowly elongated; covered with dark setae longer along upper margin and on posterior corner. Hypandrium subtriangular viewed ventrally, with two black closely set spinules apically. Phallus strongly curved, attenuated on about middle part, with dorsal projection on subapical part (lateral view), long beak-like apical opening.

**Female** (described for the first time, Fig. 2). Body length 7.2–7.9 mm, wing length 8.2–8.5 mm. Very similar to male, but mesonotum with somewhat long setae; scutal lateral spots less distinct and sometimes absent. Cercus long, slender, brown, clothed in minute setulae.

##### Material examined.

South Korea: one male, Hongcheon-gun GW, Yeongnae-ri Duchon-myeon, Mt. Baegu (37°50'39.14"N, 128°0'49.90"E), 25.V.2013, Yongjung Kwon; one male, Gangweon Prv., Mt. Obongsan (38°0'6.08"N, 127°48'22.36"E), 17.V.1981, Yongjung Kwon; one female, same locality, 19.V.1981; one female, Gangweon Prv., Mt. Seolagsan (37°50'39.14"N, 128°0'49.90"E), 19.V.1981, Yongjung Kwon.

##### Distribution.

Palaearctic: Japan (Hiroshima), South Korea.

##### Remarks.

The species has been known for a long time only from the holotype male described by Ito (1961) from Honsyû Island (Hiroshima Prefecture) of Japan. Here we record *E. sesquata* from South Korea for the first time, where this species was collected from a mountain area of Gangweon Province on dates ranging from the middle of May till the end of June. *Empis sesquata* can be readily distinguished from other species of *Xanthempis* known from the eastern Asia by its distinctive scutal pattern.

#### Empis (Xanthempis) suhi sp. n.

Taxon classificationAnimaliaDipteraHybotidae

http://zoobank.org/6442F43B-1CA9-4E5B-B296-1219403C3888

Figs 7–8 , 9–12

##### Diagnosis.

Mesoscutum with very narrow black median vitta running to base of scutellum. Occiput with narrow drop-like brown spot including ocellar tubercle. Prothoracic spiracle brown.

##### Description.

**Figures 7–8.**
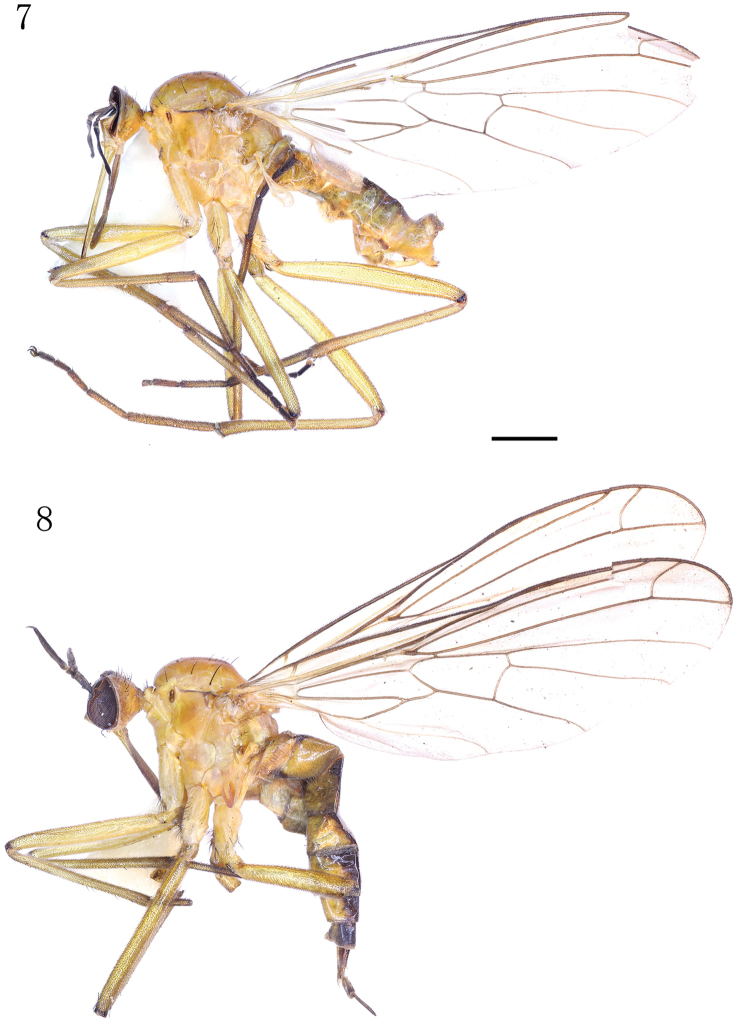
Empis (Xanthempis) suhi sp. n., habitus, lateral view. **7** Male **8** Female. Scale bar: 1 mm.

Male (Fig. 7). Body length 6.0–6.1 mm, wing length 8.2–8.8 mm. Head (Fig. 10) yellow, faintly pale greyish pollinose; occiput with narrow brown drop-like spot including ocellar tubercle. Eyes dichoptic, ommatidia equally small. Frons broad, parallel-sided, with minute dark setulae laterally. Occiput (Fig. 12) with short sparse black setae on upper part and some pale setae behind mouth-opening. Ocellar tubercle with two short proclinate oc and some minute setulae. Antennal scape and pedicel brown, postpedicel and stylus black; scape long, 4.3 times longer than wide, with some black setulae; pedicel very short, subglobular, with circle of black subapical setulae; postpedicel very long, narrow, subconical, nearly 9.5 times longer than wide; stylus very short, 0.2 times as long as postpedicel. Proboscis long, labrum 2.5–3.0 times longer than head height; palpus yellow, with scattered blackish setulae.

**Figures 9–12.**
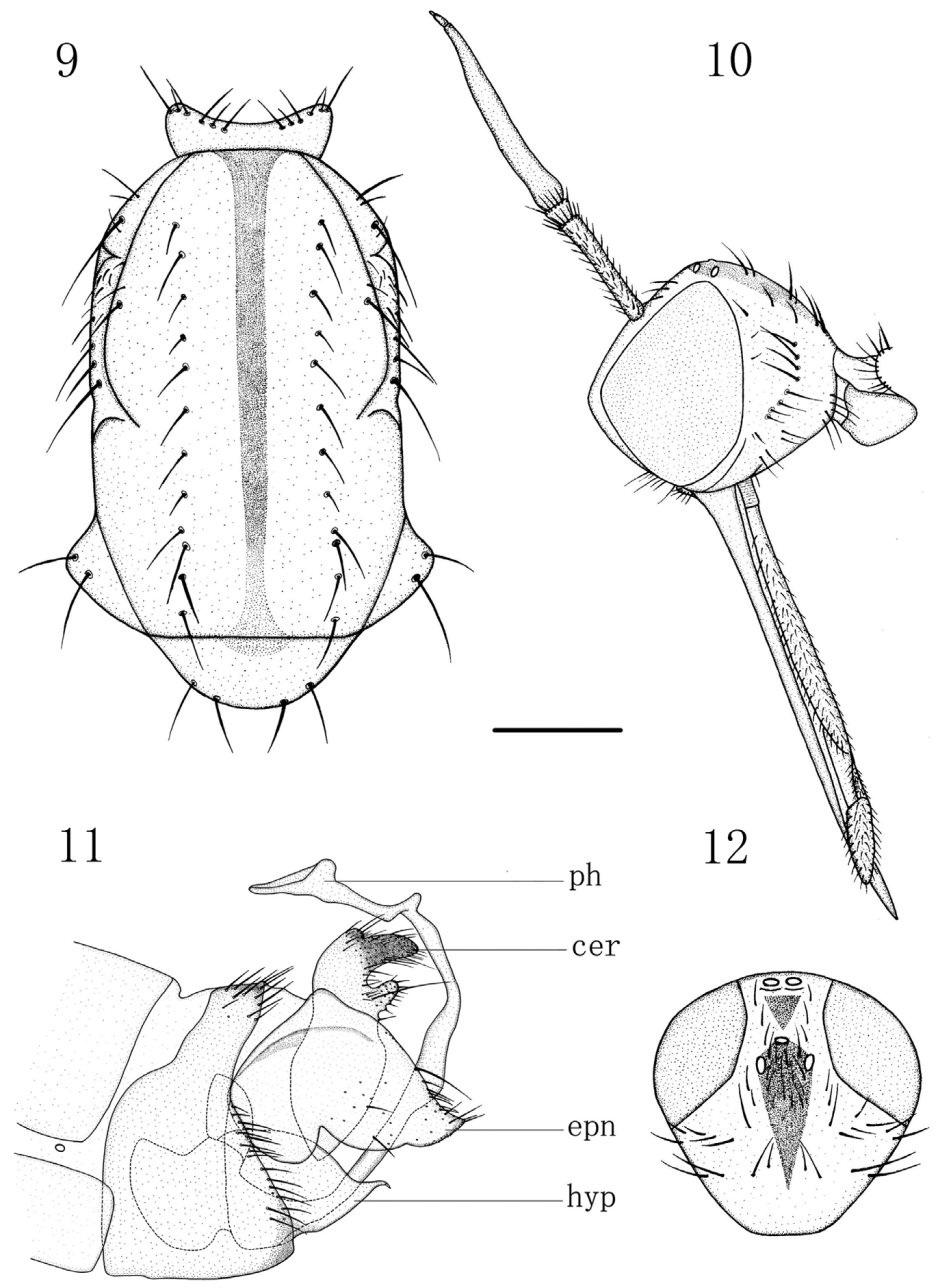
Empis (Xanthempis) suhi sp. n., male. **9** Thorax, dorsal view **10** Head, lateral view **11** Genitalia, lateral view **12** Head, dorsal view. Scale bar: 1 mm. Abbreviations: ph = phallus, cer = cercus, epn = epandrium, hyp = hypandrium.

Thorax almost entirely yellow, faintly pale greyish pollinose, with greatly reduced, only black setation; mesoscutum (Fig. 9) with very narrow black median vitta running to base of scutellum; antepronotum with brownish spot dorsally, upper border of anepisternum brown, postnotum with small indistinct brownish spot dorsally. Prothoracic spiracle brown. Prosternum bare. Proepisternum with 2–3 hair-like minute setulae on lower part. Antepronotum with several spinule-like setulae on each side. Postpronotal lobe with one moderately long inclinate ppn and 1–2 additional setulae anteriorly. Mesonotum with one moderately long presutural sa, one longest npl, one moderately long pa, four sc (apicals long, laterals short); acr absent; dc uniserial, 7–9 in row, situated far outside black median vitta, hair-like, very short (except 2–3 longer prsc); additionally, notopleuron with several setulae anteriorly. Laterotergite with numerous black setulae of different lengths. Legs yellowish, but tarsi brownish yellow to brownish; lacking prominent bristles (except circlets of short subapical setae on tibiae). Wing nearly hyaline; brownish yellow stigma long, narrow; veins dark brown; basal costal bristle black, short. Calypter yellowish, blackish fringed. Halter yellow.

Abdomen extensively yellow, but tergites broadly brownish dorsally forming uniform vitta (except tergite 8), subshiny; mostly yellowish to brownish yellow setulae longer laterally, segment 8 with black setae posteriorly. Hypopygium (Fig. 11) large, almost entirely yellow, only cerci narrowly brownish apically. Cercus rather large, with deep excision; dorsal arm long, broad, somewhat concave apically (in lateral view), ventral arm short finger-like; covered with dark minute setulae and bearing one moderately long seta on ventral arm. Epandrial lobe rather trapezoid, with upper posterior corner broadly rounded and lower posterior corner narrowly elongated; covered with dark short setae somewhat long along upper margin and on posterior corner. Hypandrium subtriangular in ventral view, with two black closely set spinules apically. Phallus strongly curved, somewhat broad near base, otherwise of more or less uniform thickness, with small dorsal tubercle closer to short beak-like apical opening.

**Female** (Fig. 2). Body length 7.1–7.2 mm, wing length 7.9–8.3 mm. Very similar to male, but postpedicel somewhat short, 8.4 times longer than wide in single specimen examined; abdominal tergites with narrower brown dorsal area. Cercus long, slender, brown, clothed in minute setulae.

##### Type material.

Holotype, male, Korea, Gangweon Pr., Mt. Seolagsan (37°50'39.14"N, 128°0'49.90"E), 29.VI.1984, Yongjung Kwon. Paratypes, one male (dissected), two females, same data as holotype.

##### Distribution.

Palaearctic: South Korea.

##### Remarks.

In the scutal pattern, the new species is similar to *E. belousovi* Shamshev, 1998 and *E. zlobini* Shamshev, 1998 known from the Russia Far East (including Sakhalin Island) and to *E. japonica* Frey, 1955 known from Hokkaido and Kuril Islands (Kunashir) (Shamshev 1998). *Empis suhi* sp. n. can be distinguished from these species as it has been given in the key.

##### Etymology.

The species is named in honor of Prof. Sang Jae Suh, Daegu in order to express our sincere thanks to him during the course of this study.

## Discussion

At the present the subgenus Xanthempis is known exclusively from the Palaearctic region with 53 described species. There are eight species known in Eastern Asia, including the Russian Far East, Mongolia, the Korean Peninsula, and Japan. The subgenus Xanthempis is recorded from South Korea for the first time with the following two species: E. (X.) sesquata (Ito) and E. (X.) suhi sp. n. *Empis sesquata* is found in both the Korean Peninsula and Japan. The new species is similar to *E. belousovi* Shamshev, 1998 and *E. zlobini* Shamshev, 1998 from Russian Far East (including Sakhalin Island), and to *E. japonica* Frey, 1955 known from Hokkaido and Kuril Islands (Kunashir) (Shamshev 1998). *Empis kovalevi* Shamshev and *E. richteri* Shamshev are distributed on the Asian continent, and *E. stercorea* Linnaeus widely spreads in the Palaearctic region. *Xanthempis* has not been reported from China yet; however, some species of the subgenus may occur in northeast China. Further collections and investigations of *Xanthempis* from these areas may provide additional data on the fauna and distribution of this subgenus in Asia.

## Supplementary Material

XML Treatment for Empis (Xanthempis) sesquata

XML Treatment for Empis (Xanthempis) suhi
